# Isoforsythiaside Attenuates Alzheimer’s Disease via Regulating Mitochondrial Function Through the PI3K/AKT Pathway

**DOI:** 10.3390/ijms21165687

**Published:** 2020-08-08

**Authors:** Chunyue Wang, Jie Hao, Xin Liu, Chenliang Li, Xuyang Yuan, Robert J. Lee, Tian Bai, Di Wang

**Affiliations:** 1 School of Life Sciences, Jilin University, Changchun 130012, China; chunyue19@mails.jlu.edu.cn (C.W.); haojie19@mails.jlu.edu.cn (J.H.); liu_xin18@mails.jlu.edu.cn (X.L.); lichenliang@jlu.edu.cn (C.L.); yuanxy1317@mails.jlu.edu.cn (X.Y.); 2 Division of Pharmaceutics, College of Pharmacy, The Ohio State University, Columbus, OH 43210, USA; lee.1339@osu.edu; 3 College of Computer Science and Technology, Jilin University, Changchun 130012, China

**Keywords:** Alzheimer’s disease, isoforsythiaside, mitochondrial apoptosis, PI3K/AKT pathway, BCL-2 family

## Abstract

Improving mitochondrial dysfunction and inhibiting apoptosis has always been regarded as a treatment strategy for Alzheimer’s disease (AD). Isoforsythiaside (IFY), a phenylethanoid glycoside isolated from the dried fruit of *Forsythia suspensa*, displays antioxidant activity. This study examined the neuroprotective effects of IFY and its underlying mechanisms. In the L-glutamate (L-Glu)-induced apoptosis of HT22 cells, IFY increased cell viability, inhibited mitochondrial apoptosis, and reduced the intracellular levels of reactive oxygen species (ROS), caspase-3, -8 and -9 after 3 h of pretreatment and 12–24 h of co-incubation. In the *APPswe*/*PSEN1dE9* transgenic (APP/PS1) model, IFY reduced the anxiety of mice, improved their memory and cognitive ability, reduced the deposition of beta amyloid (Aβ) plaques in the brain, restrained the phosphorylation of the tau protein to form neurofibrillary tangles, inhibited the level of 4-hydroxynonenal in the brain, and improved phosphatidylinositol 3-kinase (PI3K)/protein kinase B (AKT) signaling pathway-related mitochondrial apoptosis. In Aβ_1-42_-induced U251 cells, IFY relieved the mitochondrial swelling, crest ruptures and increased their electron density after 3 h of pretreatment and 18–24 h of co-incubation. The improved cell viability and mitochondrial function after IFY incubation was blocked by the synthetic PI3K inhibitor LY294002. Taken together, these results suggest that IFY exerts a protective effect against AD by enhancing the expression levels of anti-apoptosis proteins and reducing the expression levels of pro-apoptosis proteins of B-cell lymphoma-2 (BCL-2) family members though activating the PI3K/AKT pathway.

## 1. Introduction

As one of the chronic neurodegenerative disease, Alzheimer’s disease (AD) attacks more and more elderly people [1], and is characterized by memory loss and cognitive decline [2]. Two significant pathological features—senile plaques formed by insoluble beta amyloid (Aβ) deposition and neurofibrillary tangles (NFTs)—consist of the highly phosphorylated tau protein, and have been noted in AD patients’ brains [3]. The direct correlation between apoptosis and AD has been reported, which is one of the leading causes of damage to the hippocampus and cortical neurons in AD patients [4,5]. Meanwhile, mitochondria are the major organelles in cells that provide energy to meet the metabolic needs of cells [6], which contain many redox enzymes and are the source of reactive oxygen species (ROS). An excessive accumulation of ROS will change the mitochondrial membrane potential (MMP), which in turn activates apoptosis [7].

Aβ has been found in the brain mitochondria of AD patients and AD mice [8] and interacts with the mitochondrial matrix protein-binding alcohol dehydrogenase protein (ABAD), causing oxidative damage to mitochondria [9]. Furthermore, Aβ has been confirmed to induce neuronal apoptosis through the caspase cascade [10]. The Bcl-associated death promoter (BAD), a pro-apoptotic protein in the B-cell lymphoma-2 (BCL-2) family, transfers from the cytoplasm to the mitochondria responsible for the imbalance of the MMP [11], which can be prevented by phosphatidylinositol 3-kinase(PI3K)/protein kinase B (AKT) signaling via promoting its phosphorylation [11,12]. The exposure of Aβ can directly interrupt PI3K/AKT signaling in the brains of AD patients [13].

According to reports between 2000 and 2018, the number of deaths caused by AD increased by 146.2%. However, there is no effective drug that can slow or prevent the neuronal damage caused by AD [1], which emphasizes the need to search for alternative agents. Encouragingly, GV-971, an oligosaccharide extracted from marine plants, can inhibit the progression of AD by improving the intestinal microenvironment [14], and was approved for listing in China in 2019. Now, natural plants have attracted lots of researchers to screen candidate agents due to their extensive pharmacological activities and few adverse effects. Isoforsythiaside (IFY (structure presented as Figure S1A)), a phenylethanoid glycoside isolated from the dried fruit of *Forsythia suspensa* (Thunb.) Vahl (Lianqiao in Chinese) [15], is an isomer of forsythoside A (structure presented as Figure S1B). *Forsythia suspensa* (family Oleaceae), a traditional medicine in China, Korea and Japan, extracts exhibit antibacterial, antiviral, anti-inflammatory and anti-oxidant effects, and appears in the ingredients of 114 traditional Chinese medicine preparations according to the 2015 edition of the Chinese Pharmacopoeia [16,17]. Forsythoside A, one of the major component of *Forsythia suspensa*, can improve the learning ability and memory of SAMP8 mice by inhibiting oxidative stress and reducing inflammatory factors [18]. Forsythoside A acts as a neuroprotective agent by inhibiting the expression of acetylcholinesterase and caspase-3 in Aβ_25-35_-damaged PC12 cells [19], and prevents Aβ_25-35_-induced apoptosis through the cannabinoid receptor 1 (CB1R)-dependent nuclear factor-κB (NF-κB) pathway in hippocampal slices [20]. However, only one study reported the pharmacological efficacy of IFY, which is related to its antioxidant and antimicrobial activities [15]. Until now, no causal studies have confirmed the neuroprotective effect and underlying mechanisms of IFY in cells and/or animal models.

In this study, we explored the anti-AD activity of IFY in L-glutamate (L-Glu)-induced HT22 apoptotic cells, Aβ_1-42_-induced U251 apoptotic cells and the *APPswe*/*PSEN1dE9* transgenic (APP/PS1) model. Further data confirmed that the neuroprotection of IFY is related to its modulation on mitochondria apoptosis via PI3K/AKT signaling. Our data provided the experimental evidence for IFY as a potential agent in clinical applications for inhibiting or reversing AD progression.

## 2. Results

### 2.1. IFY Protected HT22 Cells against L-Glu Inducing Cell Apoptosis

**Figure 1:**
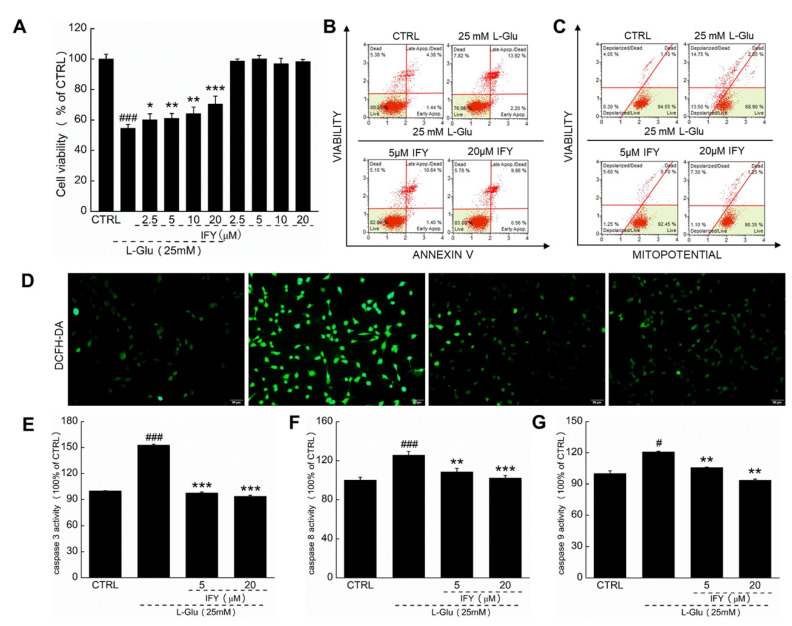
Isoforsythiaside (IFY) resisted apoptosis in HT22 cells induced by L-glutamate (L-Glu). (**A**) IFY improved the cell viability of HT22 cells exposed to L-Glu. Simultaneously, IFY showed no toxicity cultured with HT22 cells alone (*n* = 6). (**B**) IFY prevented L-Glu-induced HT22 cell apoptosis (*n* = 6). (**C**) IFY relieved the mitochondrial membrane potential (MMP) imbalance in L-Glu-damaged HT22 cells (*n* = 6). (**D**) IFY ameliorated the overaccumulation of reactive oxygen species (ROS) in HT22 cells damaged by L-Glu (magnification × 20, scale bar: 50 μm (*n* = 6)). IFY reduced the expression of (**E**) caspase-3 (*n* = 6), (**F**) caspase-8 (*n* = 6) and (**G**) caspase-9 (*n* = 6) in HT22 cells exposed to L-Glu. Data are shown as the mean ± standard error (SEM). # *p* < 0.05, ### *p* < 0.001 vs. CTRL, * *p* < 0.05, ** *p* < 0.01, *** *p* < 0.001 vs. L-Glu-exposed HT22 cells.

IFY enhanced the cell viability (*p* < 0.05 (Figure 1A)), suppressed cell apoptosis (Figure 1B), promoted cell mitochondrial function (Figure 1C) and inhibited the ROS accumulation of L-Glu-exposed HT22 cells (Figure 1D). Compared with L-Glu-damaged cells, 3-h IFY pre-treatment resulted in a >54.96%, >17.36% and >14.96% reduction on the activities of caspase-3 (*p* < 0.001 (Figure 1E)), caspase-8 (*p* < 0.01 (Figure 1F)) and caspase-9 (*p* < 0.01 (Figure 1G)). 

### 2.2. IFY Improved the Behavioral Cognitive Ability in APP/PS1 Mice

**Figure 2:**
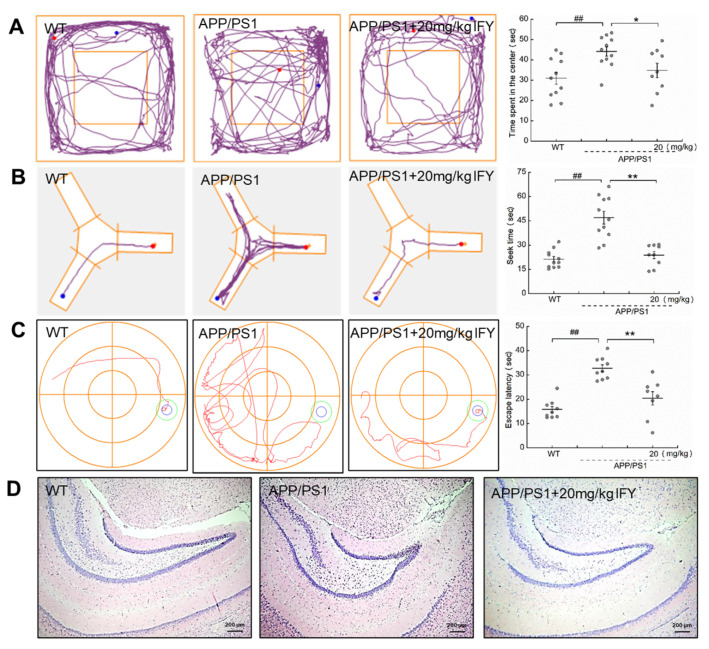
IFY ameliorated the cognitive deficiency in APP/PS1 mice. (**A**) IFY shortened the time spent in the center of the open field test. (**B**) IFY curtailed the time spent in seeking food in the Y-maze test. (**C**) IFY reduced the time spent in locating the platform of the Morris water maze (MWM) test (*n* = individual dots displayed for each group in all graphs). Data were shown as the mean ± SEM. ## *p* < 0.01 vs. wild type (WT) mice, * *p* < 0.05, ** *p* < 0.01 vs. APP/PS1 mice. (**D**) IFY showed no significant effect on mice brain tissues detected by hematoxylin–eosin (H&E) staining. (magnification × 4, scale bar: 200 μm (*n* = 3)).

Compared with the wild type (WT) mice, the memory loss and behavioral cognitive impairment were noted in APP/PS1 mice. Meanwhile, IFY significantly reduced the time spent in the central area of the open filed test (*p* < 0.05 (Figure 2A)), the searching time for food in the Y maze test (*p* < 0.01 (Figure 2B)), and the searching time for the platform in the Morris water maze (MWM) test (*p* < 0.01 (Figure 2C)). Among all groups including WT mice, APP/PS1 mice, and IFY-administered APP/PS1 mice, no significant changes were noted in brain tissues (Figure 2D) and other organs including the spleen, liver and kidney (Figure S2). 

### 2.3. IFY Improved the Pathological Status of Brain and Hippocampus in APP/PS1 Mice

**Figure 3:**
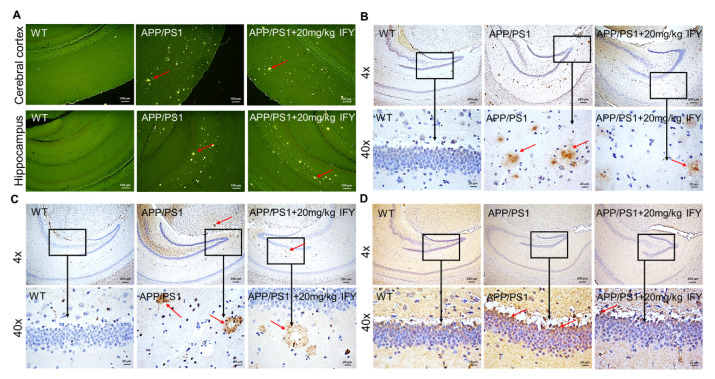
IFY improves Alzheimer’s disease (AD)-like symptoms in the brain of APP/PS1 mice. Eight-month-old APP/PS1 mice were treated with IFY (20 mg/kg, i.g.) or treated with saline (0.9%, i.g.) for 42 days. (**A**) IFY prevented the accumulation of Aβ in the cerebral cortex and hippocampus, observed via thioflavin S staining (magnification × 10, scale bar: 100 μm (*n* = 3)). (**B**) IFY reduced the expression of Aβ in the hippocampus, detected by immunohistochemical staining. (**C**) IFY suppressed the expression of p-tau in the hippocampus. (**D**) IFY decreased the expression of 4-HNE in the hippocampus studied by immunohistochemical staining. For (**B**), (**C**) and (**D**): magnification × 4, scale bar: 200 μm, magnification × 40, scale bar: 20 μm (*n* = 3).

Aβ aggregation and the hyper-phosphorylated tau protein are prominent features of AD patients [21]. Sensible plaques formed by Aβ aggregation were observed in APP/PS1 mice; however, the number of spots was reduced in IFY-treated APP/PS1 mice. The results were confirmed by thioflavin S staining (Figure 3A). The amyloid plaques in the brain cortex and hippocampus of APP/PS1 mice were strongly reduced after IFY treatment (Figure 3B). NFTs caused by hyper-phosphorylated tau are clearly visible in the brain of APP/PS1 mice, which were strongly suppressed in IFY-treated mice (Figure 3C). One of the main products of lipid peroxidation is 4-hydroxynonenal (4-HNE), which is significantly enhanced in the pathological process of AD [22]. In APP/PS1 mice, IFY significantly reduced the content of 4-HNE in the brain area (Figure 3D). 

### 2.4. IFY Regulated the Levels of Apoptosis-Related Proteins 

**Figure 4:**
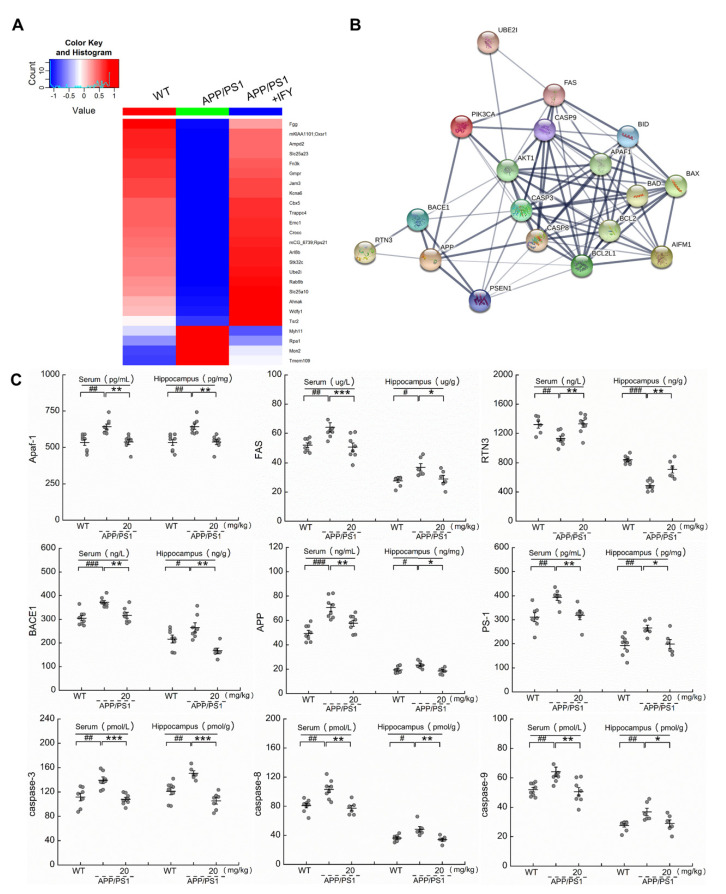
IFY regulated the expressions of apoptosis-related proteins in the serum and hippocampus. (**A**) Color-coded two-dimensional heat maps among WT, APP/PS1 and IFY-treated APP/PS1. Red represents up-regulated protein expression values, and blue represents down-regulated values (*n* = 6). (**B**) STRING protein interactive analysis for the interaction analysis of differentially expressed proteins among WT, APP/PS1 and IFY-treated APP/PS1. The thickness of the line indicates the strength of data support. (**C**) IFY ameliorated apoptosis related proteins including Apaf-1, FAS, RTN3, BACE1, APP, PS-1, caspase-3, caspase-8 and caspase-9 detected by an enzyme-linked immunosorbent assay (ELISA (*n* = individual dots displayed for each group in all graphs)). All data shown as the mean ± SEM. # *p* < 0.05, ## *p* < 0.01, ### *p* < 0.001 vs. WT mice, * *p* < 0.05, ** *p* < 0.01, *** *p* < 0.001 vs. APP/PS1 mice.

Label-free quantitative proteomics is considered a reliable tool and is used as a fairly precise measure of relative protein abundance in complex protein mixtures [23]. Compared with untreated APP/PS1 mice, IFY significantly increased the expression levels of 21 proteins and decreased the expression levels of four proteins (Table S1 (Figure 4A)), and their interactions were performed with STRINGdb (Figure 4B). Significantly changed proteins, some of which are associated with AD, especially related to anti-apoptosis, including ubiquitin-conjugating enzyme E2i (UBE2I), also known as ubiquitin-conjugating enzyme 9 (UBC9) [24], have been reported to participate in endogenous protection and can reduce the level of Aβ aggregates [25,26]. It can also inhibit apoptosis through the PI3K/AKT signaling pathway [27]. According to the results obtained from proteomic analysis, the changes of nine proteins in the serum and hippocampus of APP/PS1 mice were confirmed via an enzyme-linked immunosorbent assay (ELISA). Compared to saline-treated APP/PS1 mice, IFY reduced the level of apoptotic protease activating factor-1 (Apaf-1 (a pro-apoptotic factor that oligomerizes in response to cytochrome C release and forms apoptosome, participating in mitochondrial-mediated apoptosis [28] [*p* < 0.01])), factor-related apoptosis (FAS (a cell surface receptor inducing apoptosis in AD by transducing signals [29] [*p* < 0.05])), beta-site APP cleaving enzyme 1 (BACE1 (a key enzyme required for AD-related Aβ synthesis [30] [*p* < 0.01])), amyloid precursor protein (APP (serving as an AD biomarker related to mitochondrial damage via combining with tau [31] [*p* < 0.05])), presenilin-1 (PS-1 (leading to increasing toxicity of APP via producing a large amount of neurotoxic Aβ deposits [32] [*p* < 0.05])), caspase-3 (a well-known apoptotic executor [33] (*p* < 0.001)), caspase-8 (a protease causes mitochondrial damage by cleaving the BH3-interacting domain death agonist (BID) [34] (*p* < 0.01)) and caspase-9 (inducing neuronal apoptosis [35] (*p* < 0.05)), while increased the level of reticulon 3 (RTN3 (reducing the expression of BACE1 to inhibit the production of Aβ [36] [*p* < 0.01])), as seen in Figure 4C in the serum and hippocampus. 

### 2.5. The Activation of PI3K/AKT Signaling Is Involved in IFY-Regulated Anti-Apoptosis 

**Figure 5:**
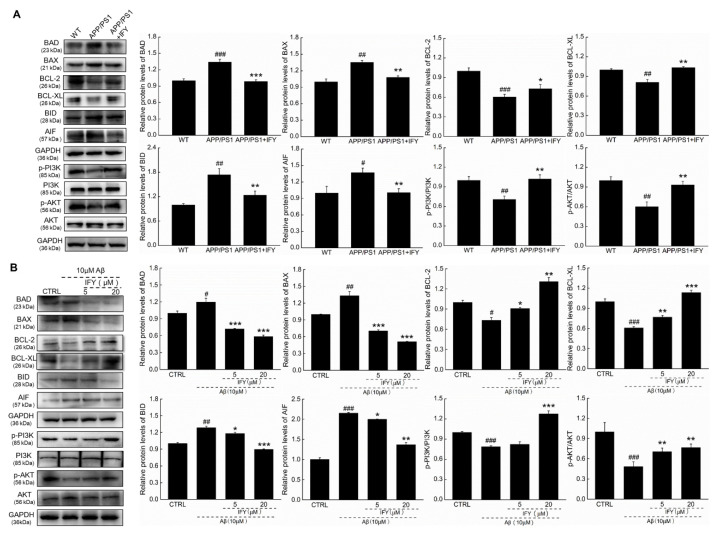
IFY regulated the expression levels of mitochondrial apoptosis-related proteins. IFY ameliorated the expression levels of apoptosis related proteins including BAD, BAX, BCL-2, BCL-XL, BID, AIF, p-PI3K and p-AKT in (**A**) the hippocampus of APP/PS1 mice and (**B**) Aβ_1-42_-exposed U251 cells. Quantification data were normalized by glyceraldehyde-3-phosphate dehydrogenase (GAPDH) and the corresponding total proteins, and are reported as the percentage of those from the corresponding CTRL (*n* = 3). Data are shown as the mean ± SEM. # *p* < 0.05, ## *p* < 0.01, ### *p* < 0.001 vs. WT mice (CTRL cells), * *p* < 0.05, ** *p* < 0.01, *** *p* < 0.001 vs. APP/PS1 mice (Aβ_1-42_-exposed U251 cells).

Aβ caused cell apoptosis by the modulation of the phosphorylated activation of P3K/AKT signaling, which is involved in cell survival, proliferation, and metabolism [37]. Compared with saline-treated APP/PS1 mice, IFY decreased the expression levels of BAD (*p* < 0.001), BCL-2-associated X protein (BAX (*p* < 0.01)), BID (*p* < 0.01) and apoptosis-inducing factor (AIF (*p* < 0.01)), and increased the expression levels of BCL-2 (*p* < 0.05), B-cell lymphoma-extra large (BCL-XL (*p* < 0.01)), p-PI3K (*p* < 0.01) and p-AKT (*p* < 0.01 (Figure 5A)). In Aβ_1-42_-exposed U251 cells, 24-h IFY incubation dose-dependently decreased the expression levels of BAD (*p* < 0.001), BAX (*p* < 0.001), BID (*p* < 0.05) and AIF (*p* < 0.05), and increased the expression levels of BCL-2 (*p* < 0.05), BCL-XL (*p* < 0.01), p-PI3K (*p* < 0.001) and p-AKT (*p* < 0.01 (Figure 5B)).

**Figure 6:**
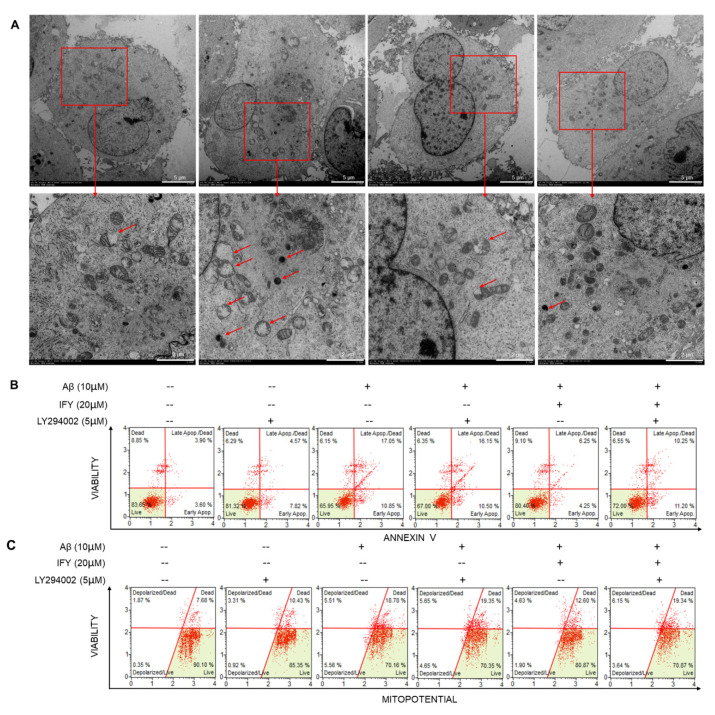
IFY resisted the mitochondrial-related apoptosis in U251 cells induced by Aβ_1-42_ which could be blocked by LY294002. (**A**) IFY improved the mitochondrial swelling, crest rupture and increased the electron density caused by Aβ_1-42_ in U251 cells observed by transmission electron microscopy (TEM (magnification × 0.7 k, scale bar: 5.0 μm [magnification × 2.0 k, scale bar: 2.0 μm] where *n* = 3)). (**B**) LY294002 undermined the anti-apoptotic effect of IFY on Aβ_1-42_-damaged-U251 cells. (**C**) LY294002 wrecked the improvement of IFY on MMP of Aβ_1-42_-damaged-U251 (*n* = 3).

A 24-h IFY exposure relieved the mitochondrial swelling, crest rupture and increased the electron density of U251 cells damaged by Aβ_1-42_ (Figure 6A). As the synthetic PI3K inhibitor, LY294002 alone failed to influence the cell viability (Figure S3), apoptosis rate (Figure 6B) and MMP levels (Figure 6C) in Aβ_1-42_-exposed U251 cells. In contrast, LY294002 co-incubation abolished the protection of IFY against Aβ_1-42_-caused cell viability reduction (*p* < 0.001 (Figure S3)), cell apoptosis (Figure 6B) and MMP dissipation (Figure 6C).

## 3. Discussion

An increased extracellular glutamate concentration causes oxidative stress, serving as one of the early events of the AD process, which is usually related to the imbalance between intracellular ROS levels and the antioxidant defense system [38,39]. An excessive production of ROS will cause mitochondrial dysfunction damage; consequently, damaged mitochondria will further accelerate the production of oxygen free radicals. The promotion of the ROS accumulation and MMP dissipation eventually leads to neuron apoptosis [40,41]. The death receptor-mediated extrinsic pathway and the mitochondrial-related intrinsic pathway are two triggering pathways of apoptosis and interfere with each other, rather than being independent of one another [42]. Among the caspase family, caspase-9 can be activated by free radicals involved in the intrinsic apoptosis, and caspase-8 helps to promote the extrinsic apoptosis [43], the two of which are responsible for the activation of caspase-3 [44]. The activated caspase-3 not only induces the cleavage of AKT, affecting the roles of Aβ in the brain [45], but also precedes and correlates with cleaved tau and NFT’s formation [46]. The activation of caspases is responsible for the accumulation of BACE and beta-secretase, thereby increasing the production of Aβ [47]. In L-Glu-damaged HT22 cells, IFY showed neuroprotection against oxidative stress-mediated mitochondrial apoptosis, and strongly reduced the levels of caspase-3, -8 and -9, suggesting its effects on the levels of Aβ and p-tau, which is similar to the previous report related to the anti-apoptotic activity of forsythoside A [19]. 

APP/PS1 double transgenic mice have been used as a typical model for AD research [48]. IFY could reduce anxiety and improve memory and spatial cognition in APP/PS1 mice according to the behavioral tests. PS1 regulates the proteolytic activity of γ-secretase leading to increased toxicity of APP via the production of a large amount of neurotoxic Aβ deposits [32]. Aβ can enter mitochondria, induce free radical production, and cause oxidative damage to mitochondria [38]. The formation of β-amyloid plaque causes the phosphorylation of the tau protein to form NFTs, which leads to the occurrence of AD [39]. Hyper-phosphorylated tau levels in AD patients’ brains are three to four times higher than those of normal brains [30]. The excessively phosphorylated tau makes the microtubules unstable and eventually leads to the death of nerve cells. Induced by cytotoxic free radicals, 4-HNE is a product of membrane lipid peroxidation which can promote apoptosis by inducing mitochondrial damage and activating caspase-3, and increase the basal levels of tau protein phosphorylation [49,50]. IFY reduced Aβ deposition, nerve fiber tangles formed by phosphorylated tau proteins, and the levels of 4-HNE in the brain of APP/PS1 mice.

Based on the proteomic analysis, IFY significantly up-regulated UBE2I expression in the hippocampus of APP/PS1 mice. The up-regulation of UBE2I expression leads to an increase in the APP sulfonating level and a decrease in the Aβ protein aggregation level [51]. The overexpression of UBE2I will cause a significant increase in the phosphorylation of the AKT protein and resist apoptosis via the PI3K/AKT signaling pathway [27]. BACE1 is an important enzyme that cleaves APP at the β-site to produce CTFβ, which is then cleaved by γ-secretase to form Aβ [30]. The free form of RTN3 interacts with BACE1 and inhibits BACE1-mediated APP processing to form Aβ [52], thereby reducing the formation of amyloid plaque and delaying the AD process. Additionally, RTN3 causes BCL-2 the translocate to mitochondria, promoting mitochondrial functions and cell survival [53]. 

Accordingly, apoptosis is partially coordinated by the caspase family, which can be regulated by members of the BCL-2 family [54]. The overexpression of BCL-2 limits the activation of caspase-9 by interacting with the Apaf-1, weakening the processing of APP and the caspase cleavage of tau, and thus reducing the deposition of Aβ and the formation of NFTs [55]. The ratio of BAX/BCL-2 is usually used to measure the survival status of cells [56]. BCL-XL strictly regulates the survival of immature neurons during the development of the nervous system. As the substrate of caspase-8, the truncated BID transfers to the mitochondria and promotes AIF and cytochrome C’s release from mitochondria [57]. AIF is cleaved by cysteine protease to form tAIF, which enters the nucleus responsible for chromatin condensation and DNA degradations [58]. The release of cytochrome C activates apoptosome and Apaf-1, required for mitochondrial apoptosis [59,60]. Apaf-1 is a key molecule in the mitochondrial pathway of apoptosis. After cytochrome C is released from the mitochondria, apaf-1 aggregates to form apoptosome, subsequently activating caspase-9 and caspase-3, and finally triggering apoptosis [28]. Furthermore, unphosphorylated BAD can enter mitochondria and interact with BCL-XL to exhibit the pro-apoptotic effect [11,12]. The phosphorylated AKT, activated by PI3K, can promote the phosphorylation of BAD at the Ser 136 site, which loses the ability to bind to BCL-XL, achieving anti-apoptotic effects [11]. The activation of PI3K/AKT signaling can decrease tau protein hyper-phosphorylation at Ser 404 and Ser 413 via inhibiting GSK-3β activity through activating the phosphorylation of GSK-3β at serine 9 [61], and enhancing the function of BCL-2 to promote cell survival [62,63]. Our present data confirmed that IFY can suppress the neuron apoptosis via regulating the expression levels of BCL-2 family members through activating PI3K/AKT signaling. 

In summary, to our knowledge, this study is the first confirm the neuroprotective effects of IFY in L-Glu-damaged HT22 cells, Aβ_1-42_-induced apoptotic U251 cells, and APP/PS1 mice. IFY relieves mitochondrial apoptosis via enhancing the expression levels of anti-apoptosis proteins and reducing the expression levels of pro-apoptosis proteins of BCL-2 family members through activating PI3K/AKT signaling. Our data provided the experimental evidence for further investigating IFY as a potential agent in clinical applications for inhibiting or reversing AD progression. 

## 4. Materials and Methods

### 4.1. Materials and Reagents

IFY (Cas No.: 1357910-26-9; purity ≥ 98%), 3-(4,5-dimethylthiazole-2-yl)-2,5-diphenyl tetrazolium bromide (MTT) and 4% glutaraldehyde (R20513) were purchased from Shanghai Yuanye Biological Technology Co., Ltd. (Shanghai, China); Aβ_1-42_ oligomers (purity ≥ 95%) were obtained from Gill Biochemical Co., Ltd. (Shanghai, China), the preparation and identification process of Aβ1-42 oligomers is similar to the reported study [64]. In short, Aβ_1-42_ was dissolved in hexafluoro-2-propanol (HFIP) and then HFIP was removed by evaporation in a vacuum to form a peptide film. The samples were dissolved in dimethyl sulfoxide (DMSO), and then diluted with Ham’s F12 medium without phenol red and incubated overnight at 4 °C. After centrifugation at 14,000× *g* for 10 min, the supernatant were collected and freeze-dried; fetal bovine serum (FBS) was purchased from Kang Yuan Biology (Tianjin, China); LY294002 (GC15485) was purchased from GlpBio (Montclair, CA, USA); DMSO (DH105-2) was obtained from Beijing Dingguo Changsheng Biotechnology Co., Ltd. (Beijing, China); dulbecco’s modified Eagle’s medium (DMEM), streptomycin, penicillin and the Pierce™ BCA Protein Assay Kit (23225) were acquired from Thermo Fisher Scientific (Waltham, MA, USA); Annexin V and Dead Cell Reagent (MCH100105) and an electrochemiluminescence kit were purchased from EMD Millipore Corp. (Billerica, MA, USA); 5, 5′, 6, 6′-tetrachloro-1, 1′, 3, 3′-tetraethyl-imidacarbocyanine iodide (JC-1 (Catalog: BB-4105-20T)); caspase-3 (BB-4106), caspase-8 (BB-4107) and caspase-9 (BB-4108) were obtained from BestBio (Shanghai, China); 2, 7-dichlorofuorescin diacetate (DCFH-DA (E004-1-1)) was acquired from Nangjing Jiancheng Bioengineering Institute (Nanjing, China); L-Glu (G8415), osmium tetroxide, sodium deoxycholate (SDC), radio immunoprecipitation assay (RIPA), phenylmethanesulfonyl fluoride (PMSF), protease inhibitor cocktail and bovine serum albumin (BSA) were purchased from Sigma-Aldrich (St. Louis, MO, USA); caspase-3 (F9179-A), caspase-8 (KT9229-A), caspase-9 (KT9243-A), RTN3 (KT9238-A), BACE1 (KT9248-A), APP (KT2824-A), PS-1 (KT9233-A), Apaf-1 (KT9243-A) and FAS (KT9230-A) were acquired from Jiangsu Kete Biotechnology Co., Ltd. (Yancheng, Jiangsu, China); tau (phospho S396 (ab109390)), 4-HNE (ab46545), BAD (ab32445), BAX (ab32503), BCL-XL (ab32370), BID (ab62469), PI3K p85 (phospho Y607 (ab182651)), AKT (phospho T450 (ab108266)) and AKT (ab200195) were purchased from abcam (Cambridge, MA, USA); Aβ_1-42_ (bs-0107R), BCL-2 (bsm-33047M) and AIF (bs-0037R) were purchased from Bioss Antibodies (Beijing, China); PI3K (4292s) was acquired from Cell Signaling Technology (Beverly, MA, USA); glyceraldehyde-3-phosphate dehydrogenase (GAPDH (E-AB-20059)), goat anti-rabbit IgG (H+L (E-AB-1003)) and goat anti-mouse IgG (H+L (E-AB-1001)) were acquired from Elabscience Biotechnology Co., Ltd. (Wuhan, China); polyvinylidene difluoride membranes was purchased from GE Healthcare Life Science (Beijing, China).

### 4.2. Cell Culture 

HT22 cells, a mouse hippocampal neuronal cell line (No. BNCC337709), and U251 cells, a human astroglioma cell line (No. BNCC337874 (both from BeNa Culture Collection, Beijing, China)), were grown in DMEM supplemented with 10% FBS, 1% 100 μg/mL streptomycin and 100 units/mL penicillin at 37 ΰC in the presence of 5% CO_2_. 

### 4.3. Cell Viability Assay 

HT22 cells (8 × 10^3^ cells/well) were seeded into a ninety-six well plate and incubated at 37 °C for 12 h. HT22 cells were pre-incubated with IFY at doses of 2.5 μM, 5 μM, 10 μM, and 20 μM for 3 h, followed with a 24-h co-exposure to 25 mM of L-Glu at 37 °C. 

U251 cells (8 × 10^3^ cells/well) were seeded into a ninety-six well plate and incubated at 37 °C for 12 h. U251 cells were pre-incubated with 20 μM of IFY for 3 h, following with 24-h co-exposure to 10 μM of Aβ_1-42_ and/or 5 μM of LY294002 for another 24 h. 

After incubation, MTT (5 mg/mL) was added to each well and incubated for another 4 h at 37 °C in darkness. After discarding the supernatant, 100 μL of DMSO was added and the absorbance at 490 nm was analyzed using a Gen5^TM^ Microplate Reader (Synergy 4, Omega Bio-Tek, Inc., Norcross, GA, USA). 

### 4.4. Apoptosis and MMP Detection 

HT22 cells (2 × 10^5^ cells/well) were seeded into a six-well plate and incubated at 37 °C for 12 h. Cells were pre-treated with IFY at doses of 5 μM and 20 μM for 3 h, and then co-exposed to 25 mM of L-Glu for 24 h (apoptosis assay) or 18 h (MMP detection). U251 cells (2 × 10^5^ cells/well) were seeded into a six-well plate and incubated at 37 °C for 12 h. Cells were pre-treated with 20 μM of IFY for 3 h, and then co-exposed to 10 μM of Aβ_1-42_ and/or 5 μM of LY294002 for another 24 h (apoptosis assay) or 18 h (MMP detection). The cells were collected and resuspended using phosphate buffer saline (PBS) containing 1% FBS. Cells were stained with Annexin V and Dead Cell Reagent for 20 min (apoptosis assay), or JC-1 for 20 min (MMP detection) at 37 °C in darkness. A Muse^TM^ Cell Analyzer (EMD Millipore, Billerica, MA, USA) was used to analyze the apoptosis rate and MMP changes in cells. 

### 4.5. ROS Fluorescence Detection 

HT22 cells (1.5 × 10^5^ cells/well) were seeded into a six-well plate and incubated at 37 °C for 12 h. Cells were pre-treated with IFY at doses of 5 μM and 20 μM for 3 h, and then co-exposed to 25 mM of L-Glu for 12 h. Then, the cells were stained with a 10 μM of DCFH-DA probe for 20 min in darkness. The fluorescence intensity was analyzed under a fluorescence microscope (CKX31, Olympus, Tokyo, Japan).

### 4.6. Caspase Detection

HT22 cells (2 × 10^5^ cells/well) were seeded into a six-well plate and incubated at 37 °C for 12 h. Cells were pre-treated with IFY at doses of 5 μM and 20 μM for 3 h, and then co-exposure to 25 mM of L-Glu for 24 h. Treated cells were collected, lysed and centrifuged at 1000 rpm for 10 min. The activities of caspase-3, caspase-8 and caspase-9 were analyzed via commercial kits. 

### 4.7. Transmission Electron Microscopy (TEM)

U251 cells (2 × 10^5^ cells/well) were seeded into a six-well plate and incubated at 37 °C for 12 h. Cells were pre-treated with 5 μM and 20 μM of IFY for 3 h, and then co-exposed to 10 μM of Aβ_1-42_ for another 24 h. Treated cells were washed and fixed with 4% glutaraldehyde at 4 °C for 12 h, then post-fixed in 1% osmium tetroxide at 4 °C for 2 h. After that, cells were dehydrated in ethanol, acetone and propylene oxide. Subsequently, cells were embedded in SPI-PON 812, and then the ultra-thin sections were obtained using an ultra-thin microtome (EM UC7, Leica Microsystems, Wetzlar, Germany). After staining with uranyl acetate and lead citrate, the ultrastructure of U251 cells was observed via a TEM (H-7650, HITACHI, Tokyo, Japan). 

### 4.8. Animal Experimental Protocol 

The protocol and procedures employed were conducted in accordance with the Animal Research: Reporting In Vivo Experiments (ARRIVE) guidelines and were ethically reviewed and approved by the Institutional Animal Ethics Committee of Jilin University (License No.: SY201904010). All mice were purchased from Nanjing Biomedical Research Institute of Nanjing University (Nanjing, China (SCXK (SU) 2015-0001)), and raised at a room with a temperature of 23 ± 2 °C and a humidity of 40–60% with a 12-h:12-h light/dark cycle, and given free water and food. Twenty-four B6C3-Tg (*APPswe*/*PSEN1dE9*)/Nju double transgenic male mice (genotype: (*APPswe*) T, (*Psen1*) T (APP/PS1, respectively [8 months, 42–49 g])) were randomly divided into two groups orally revived with normal saline (*n* = 12) and 20 mg/kg of IFY (*n* = 12) for 42 days. Another twelve wild type male mice (genotype: (*APPswe*) W, (*Psen1*) W (WT (8 months, 38–47 g)) were orally revived with normal saline for 42 days. The behavioral training began on the 36th day of the whole experiment. After the last behavioral test, the mice were euthanized by injecting barbiturate sodium. The serum together with tissues, including brain, liver, spleen and kidney tissues, were collected for a biochemical and pathological analysis (Figure S4). 

### 4.9. Behavioral Experiments

#### 4.9.1. The Open Field Test 

The open field test is widely used in animal psychology to detect behavioral anxiety [65]. The experiment was performed after 30 days of administration. The open field device is 50 × 50 cm divided into the central area of 25 × 25 cm and the surrounding area. The movement trajectory of mice and the time spent in the central area were recorded by a camera for 5 min and analyzed via a software (Any-maze^TM^, Stoelting Co., Chicago, IL, USA). To avoid a previous mouse’s information influencing the results of another mouse, the apparatus must be cleaned before testing of each mouse.

#### 4.9.2. Y-Maze Test 

Y-maze tests are commonly used to investigate age-related work and spatial reference memory deficits [66]. The Y-maze is composed of three arms (47 cm × 16 cm × 46 cm for each one) with three equal divisions and a central area. There is a food supply device at the end of one arm. The training of the Y-maze began on the 38th day. After 3 days of training, the mice fasted for 12–18 h and were put into the arm for searching the food supplied at the end of one of the other two arms. The movement trajectory and searching time were recorded by a camera and analyzed via software (Any-maze^TM^, Stoelting Co., Chicago, IL, USA). 

#### 4.9.3. MWM Test 

The MWM test is a classic task for testing spatial memory, which is entirely dependent on hippocampal function [67]. The MT-200 Water Labyrinth Video Tracking Analysis System (S7200) with a circular pool with a 100 cm diameter was used for the MWM test. The training of the MWM began on the 43rd day. After 4 days of training (60 s for each mouse), the experimental mice were put into the circular pool filled with a depth of 40 cm of water (24 ± 2 °C), containing titanium dioxide, which is 0.5 cm higher than the platform. The movement trajectory and the time spent for searching the platform were recorded by a camera within 60 s and analyzed by software (watermaze 2.0., TECHMAN Software Co., Ltd., Chengdu, China).

### 4.10. Label-Free Quantitative Proteomics 

The hippocampal tissues were homogenized in RIPA buffer containing 2% PMSF and a 1% protease inhibitor cocktail. After quantification with the Pierce™ BCA Protein Assay Kit, proteins were precipitated with acetone. A total of 200 μL of 100 mM ammonium bicarbonate containing 1% SDC was added to re-dissolve the protein pellet. Values of 5 mM tris (2-carboxyethyl) phosphine and 10 mM iodoacetamide were added to alkylate the reduced disulfide bond, and then enzymatically digested with 2 μg trypsin. Trifluoroacetic acid was added to the mixed sample to precipitate SDC. After desalting the peptides, they were separated by the nano-UPLC (EASY-nLC1200, Thermo Fisher Scientific, Waltham, MA, USA) and detected using Q-Exactive mass spectrometry (Thermo Finnigan, Silicon Valley, CA, USA). Raw MS files were processed with MaxQuant (Version 1.5.6.0, Max Planck Institute, Munich, Germany). The quantification type was label-free quantification which was calculated by a label-free, intensity-based absolute quantification approach. Each group of samples contained hippocampal tissues of 6 mice.

When the ratio of protein between the two groups was greater than 1.5 or less than 0.66, they were considered to be proteins with significant differences. The cluster heat map and protein interaction analysis were executed.

### 4.11. ELISA

The collected hippocampus tissues were homogenized with normal saline, and the protein concentration was detected via a BCA kit. The levels of caspase-3, caspase-8, caspase-9, RTN3, BACE1, APP, PS-1, Apaf-1 and FAS in the serum and the hippocampus were analyzed by commercial kits according to the structures.

### 4.12. Hematoxylin-Eosin (H&E) Staining, Immunochemistry and Thioflavin S Staining

The collected brains and organ tissues, including spleen, liver and kidney tissues, were fixed with a 4% formalin solution at 25 °C for 24 h, and then dehydrated with 30%, 50%, 70%, 80%, 95% and 100% ethanol, washed in xylene, embed in paraffin and cut into 5 μm thick sections. All slides, including brain, spleen, liver and kidney samples, were gradient hydrated with 100%, 95%, 80%, 70% and 50% ethanol and distilled water in order, and the sections were stained with H&E. 

The brain slides were dewaxed, hydrated and boiled in 10 mM of sodium citrate buffer for 10 min, then cooled at 25 °C for 30 min. After incubating in 3% hydrogen peroxide for 10 min, the sections were blocked with normal 10% goat serum at 25 °C for 30 min, and then treated with primary antibodies against Aβ_1-42_ (dilution of 1:800), tau (phospho S396 (dilution of 1:4000)) and 4-HNE (dilution of 1:200) at 4 °C overnight, followed by an incubation with goat anti-rabbit IgG (H+L (dilution at 1:5000) secondary antibody at 25 °C for 1 h. The peroxidase conjugate was stained for 5 min at 25 °C using a 5% diaminobenzidine tetrahydrochloride solution as a color reagent and hematoxylin, and then, the slides were observed using an optical microscope (Olympus Corporation, Tokyo, Japan).

The brain slices were soaked in xylene for 10 min twice, 100% ethanol for 10 min twice, 95%, 90%, 80%, and 70% ethanol for 5 min in sequence, and deionized water for 30 s. Then the slides were exposed to 0.3% thioflavin S at room temperature for 8 min. After washing with 50% alcohol for 3 times, the slides were observed under a fluorescence microscope (BX51, Olympus, Tokyo, Japan).

### 4.13. Western Blot 

U251 cells (2×10^5^ cells/well) were seeded into a six-well plate and incubated at 37 °C for 12 h. Cells were pre-treated with 20 μM of IFY for 3 h, and then co-exposed to 10 μM of Aβ_1–42_ for another 24 h. Protein samples were collected from hippocampus tissues or U251 cells via lysing in RIPA buffer containing 2% PMSF and a 1% protease inhibitor cocktail. After detecting the protein concentration via a BCA kit, 40 μg of protein were separated using sodium dodecyl sulfate-polyacrylamide gel electrophoresis, and then transferred to polyvinylidene difluoride membranes. The membranes were blotted by 5% BSA at 4 °C for 4 h, and then incubated with primary antibodies at 4 °C for 12 h as follows: BAD (dilution 1:2000 (23 kDa)), BAX (dilution 1:2000 (21 kDa)), BCL-XL (dilution 1:2000 (26 kDa)), BID (dilution 1:2000 (28 kDa)), PI3K p85 (phospho Y607 (dilution 1:2000 [85 kDa])), AKT (phospho T450 (dilution 1:2000 [56 kDa])), AKT (dilution 1:2000 (56 kDa)), BCL-2 (dilution 1:1000 (26 kDa)), AIF (dilution 1:2000 (57 kDa)), PI3K (dilution 1:1000 (85 kDa)), glyceraldehyde-3-phosphate dehydrogenase (GAPDH (dilution 1:2000 [36 kDa])), followed with an incubation on horseradish peroxidase-conjugated secondary antibody at a dilution of 1:5000 including goat anti-rabbit IgG (H+L) and goat anti-mouse IgG (H+L) at 25 °C for 4 h. An electrochemiluminescence kit was used to visualize the protein bands under the imaging system (Tanon 5200, Tanon Science & Technology Co., Ltd., Shanghai, China). The quantitation of the results was accomplished by Image J software (National Institutes of Health, Bethesda, MD, USA).

### 4.14. Statistical Analysis. 

Data are expressed as the mean ± standard error (SEM). The statistical significance was determined using SPSS 16.0 software and using a one-way analysis of variance, then post hoc multiple comparisons were performed (Dunn test). *p* < 0.05 was considered to be statistically significant difference.

## Supplementary Materials

Supplementary Materials can be found at https://www.mdpi.com/1422-0067/21/16/5687/s1.

## Abbreviations

AD	Alzheimer’s disease
AKT	protein kinase B
Apaf-1	apoptotic protease activating factor-1
APP	amyloid precursor protein
APP/PS1	APPswe/PSEN1dE9 transgenic
Aβ	beta amyloid
BACE1	beta-site APP cleaving enzyme 1
BAD	Bcl-associated death promoter
BAX	BCL-2-associated X protein
BCL-2	B-cell lymphoma-2
BCL-XL	B-cell lymphoma-extra-large
BID	BH3-interacting domain death agonist
DMEM	Dulbecco’s modified Eagle’s medium
DMSO	dimethyl sulfoxide
ELISA	enzyme-linked immunosorbent assay
FAS	factor related apoptosis
FBS	fetal bovine serum
GAPDH	glyceraldehyde-3-phosphate dehydrogenase
HFIP	hexafluoro-2-propanol
H&E	hematoxylin-eosin
IFY	Isoforsythiaside
L-Glu	L-glutamate
MMP	mitochondrial membrane potential
MWM	Morris water maze
NFTs	neurofibrillary tangles
PBS	phosphate buffer saline
PI3K	phosphatidylinositol 3-kinase
PMSF	phenylmethanesulfonyl fluoride
PS-1	Presenilin-1
RIPA	radio immunoprecipitation assay
ROS	reactive oxygen species
RTN3	reticulon 3
SDC	sodium deoxycholate
TEM	transmission electron microscopy
WT	wild type
4-HNE	4-hydroxynonenal
