# Supplementary material for: Isoforsythiaside Attenuates Alzheimer’s Disease via Regulating Mitochondrial Function Through the PI3K/AKT Pathway
Source: Int J Mol Sci. 2020 Aug 8;21(16):5687. doi: 10.3390/ijms21165687 (PMC7460834; doi:10.3390/ijms21165687)
Supplement: Supplementary file 1 [file ijms-21-05687-s001.pdf]

# Supplemental Materials

## Table List

**Table S1** Proteins with significantly different expression levels in proteomics.

| Number | Gene. names | Unique. peptides | fc. APP/PS1 - WT | fc. APP/PS1+IFY -APP/PS1 |
|---|---|---|---|---|
| **Upregulated proteins by isoforsythiaside (IFY) (Number: 21)** | | | | |
| 1 | Fgg | 2 | 0.41 | 1.99 |
| 2 | mKIAA1101; Oxsr1 | 2 | 0.23 | 3.81 |
| 3 | Ampd2 | 7 | 0.19 | 4.52 |
| 4 | Slc25a23 | 5 | 0.09 | 9.46 |
| 5 | Fn3k | 4 | 0.12 | 7.64 |
| 6 | Gmpr | 4 | 0.16 | 5.83 |
| 7 | Jam3 | 4 | 0.12 | 8.25 |
| 8 | Kcna6 | 2 | 0.39 | 2.56 |
| 9 | Cbx5 | 3 | 0.22 | 5.01 |
| 10 | Trappc4 | 3 | 0.26 | 4.20 |
| 11 | Emc1 | 7 | 0.19 | 5.91 |
| 12 | Crocc | 4 | 0.13 | 8.59 |
| 13 | mCG_6739; Rps21 | 2 | 0.20 | 5.90 |
| 14 | Arl8b | 3 | 0.08 | 15.23 |
| 15 | Stk32c | 4 | 0.22 | 5.50 |
| 16 | Ube2i | 2 | 0.35 | 3.45 |
| 17 | Rab9b | 3 | 0.27 | 4.67 |
| 18 | Slc25a10 | 4 | 0.65 | 1.63 |
| 19 | Ahnak | 6 | 0.22 | 6.81 |
| 20 | Wdfy1 | 6 | 0.39 | 3.72 |
| 21 | Tsr2 | 3 | 0.41 | 4.53 |
| **Downregulated proteins by IFY (Number: 4)** | | | | |
| 1 | Myh11 | 9 | 1.53 | 0.52 |
| 2 | Rpa1 | 2 | 35.80 | 0.03 |
| 3 | Mon2 | 5 | 2.41 | 0.60 |
| 4 | Tmem109 | 2 | 3.72 | 0.54 |

fc. APP/PS1-WT: the ratio of protein between saline-treated APP/PS1mice and WT mice;

fc. APP/PS1+IFY-APP/PS1: the ratio of protein between IFY-treated APP/PS1 mice and saline-treated APP/PS1 mice.

# Figure List

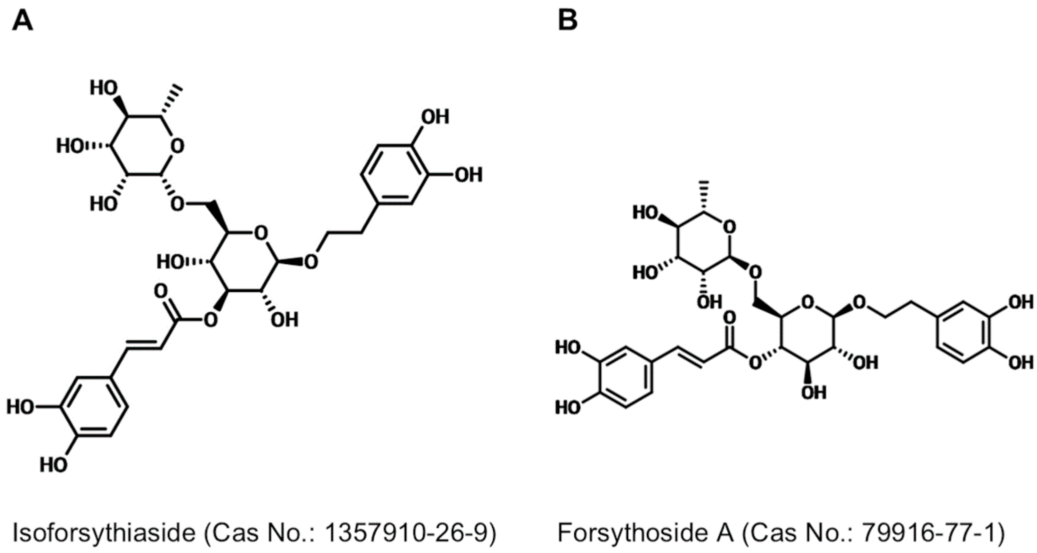


**Figure S1.** Chemical structure of (A) isoforsythiaside (Cas NO.: 1357910-26-9) and (B) forsythoside A (Cas No.: 79916-77-1).

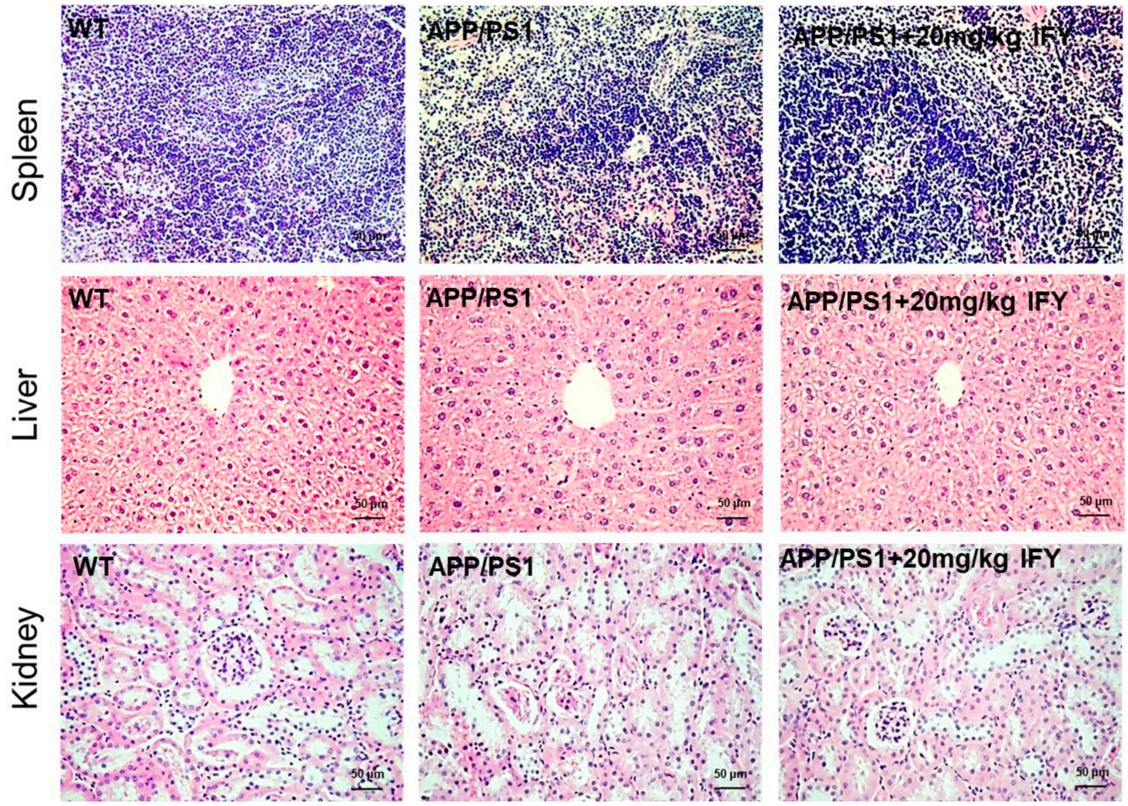


**Figure S2.** H & E staining of spleen, liver and kidney tissues (magnification x 20, scale bar: 50 μm; n=3).

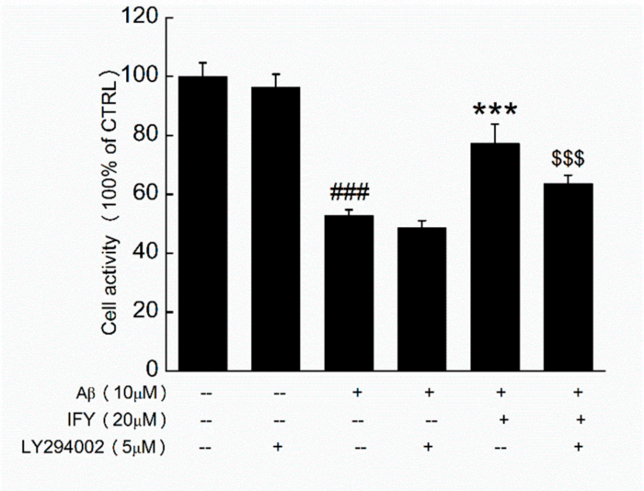


**Figure S3.** Cell viability of U251 cells exposed to $A\beta_{1-42}$ and LY294002 tested by MTT (n=6). IFY significantly increased the viability of $A\beta_{1-42}$-treated U251 cells, however, LY294002 blocked down the protective effect of IFY. ###P<0.001 vs. CTRL cells, ***P<0.001 vs. $A\beta_{1-42}$-exposed U251 cells, $$$P<0.001 vs. IFY and $A\beta_{1-42}$-exposed U251 cells.

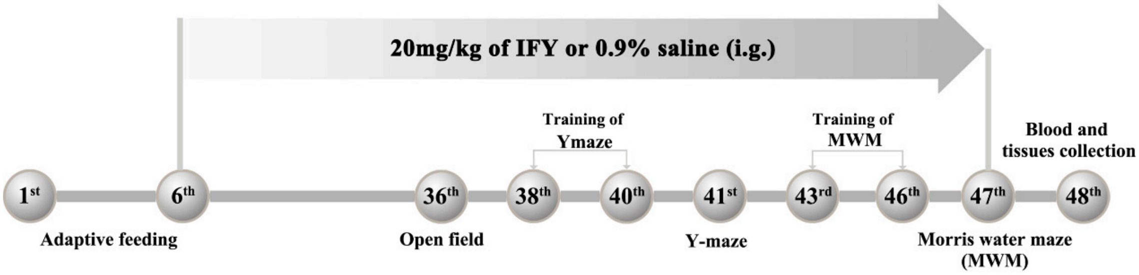


**Figure S4.** Schematic flow chart of animal experiment.
